# Para-aortic retroperitoneal robotic assisted laparoscopic schwannoma excision

**DOI:** 10.1093/jscr/rjag447

**Published:** 2026-06-10

**Authors:** Ari Solomon, Diego Monasterio Oliver, Karim Jreije

**Affiliations:** Community Memorial Hospital System, 147 N Brent Street, Ventura, CA 93003, United States; Department of Trauma, Ventura County Medical Center, 300 Hillmont Ave, Ventura, CA 93003, United States; Department of Trauma, Ventura County Medical Center, 300 Hillmont Ave, Ventura, CA 93003, United States

**Keywords:** schwannoma, retroperitoneal mass, minimally invasive surgery, para-aortic mass

## Abstract

Schwannomas are benign tumors primarily managed with surgical excision. Patient positioning, port placement, and adjuvant modalities are critical to achieve successful and safe surgical operations. In this case, a left ureteral stent was placed and the patient then placed in right lateral decubitus position. Four robotic ports were placed midline, evenly spaced between the falciform ligament and pubis. A modified mattox maneuver was executed, exposing the mass and allowing for complete excision without damage to surrounding structures. An enbloc complete excision of schwannoma without injury to surrounding structures was completed. This case highlights the importance of preoperative planning, specifically patient/port positioning and stent placement.

## Introduction

Schwannomas are benign tumors originating from schwann cells that can form along any myelinated nerve, typically presenting in the face. Management is primarily surgical excision when compression to surrounding structures leads to symptoms. This case describes a unique and rare periaortic retroperitoneal 4 cm schwannoma that was laparoscopically excised in completion.

Schwannomas are benign tumors originating from schwann cells, which form the myelin sheath surrounding peripheral nerves and allow for neuronal protection and signal transmission. These tumors are typically slow-growing and can arise along any nerve containing Schwann cells [1]. They typically have low malignant potential and are made exclusively of schwann cells [2]. Schwannomas are usually solitary and sporadic, but are also seen in genetic syndromes including neurofibromatosis and schwannomatosis [3]. Clinically, they often present as painless, slow-growing masses, but symptoms can arise from the mass effect leading from compression of adjacent structures. They are typically found in the head and neck, and are often found abutting cranial nerves, but they can present along any nerves containing schwann cells [4].

Histologically, schwannomas are characterized by a strong expression of S-100 protein and are composed predominantly of Schwann cells [5, 6]. Imaging, particularly magnetic resonance imaging (MRI), and biopsy are the gold standard for diagnosis, revealing well-circumscribed, often enhancing lesions [1, 7–11].

Management is primarily surgical, aiming for complete resection while preserving neurological function. Radiotherapy may be considered for residual or unresectable tumors. Recurrence after complete excision is uncommon [1].

## Case report

**Figure 1:**
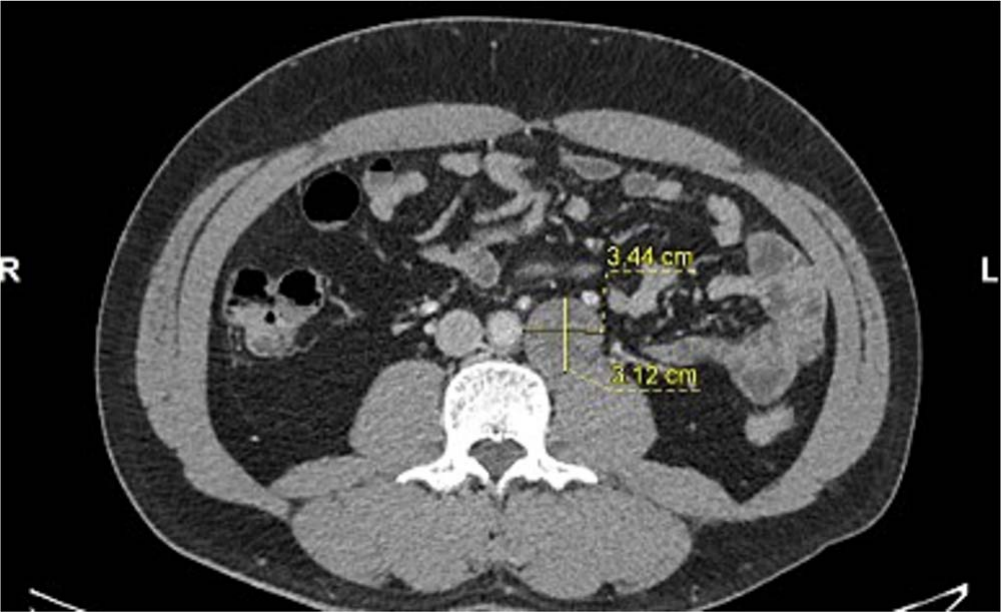
Initial CT transverse.

**Figure 2:**
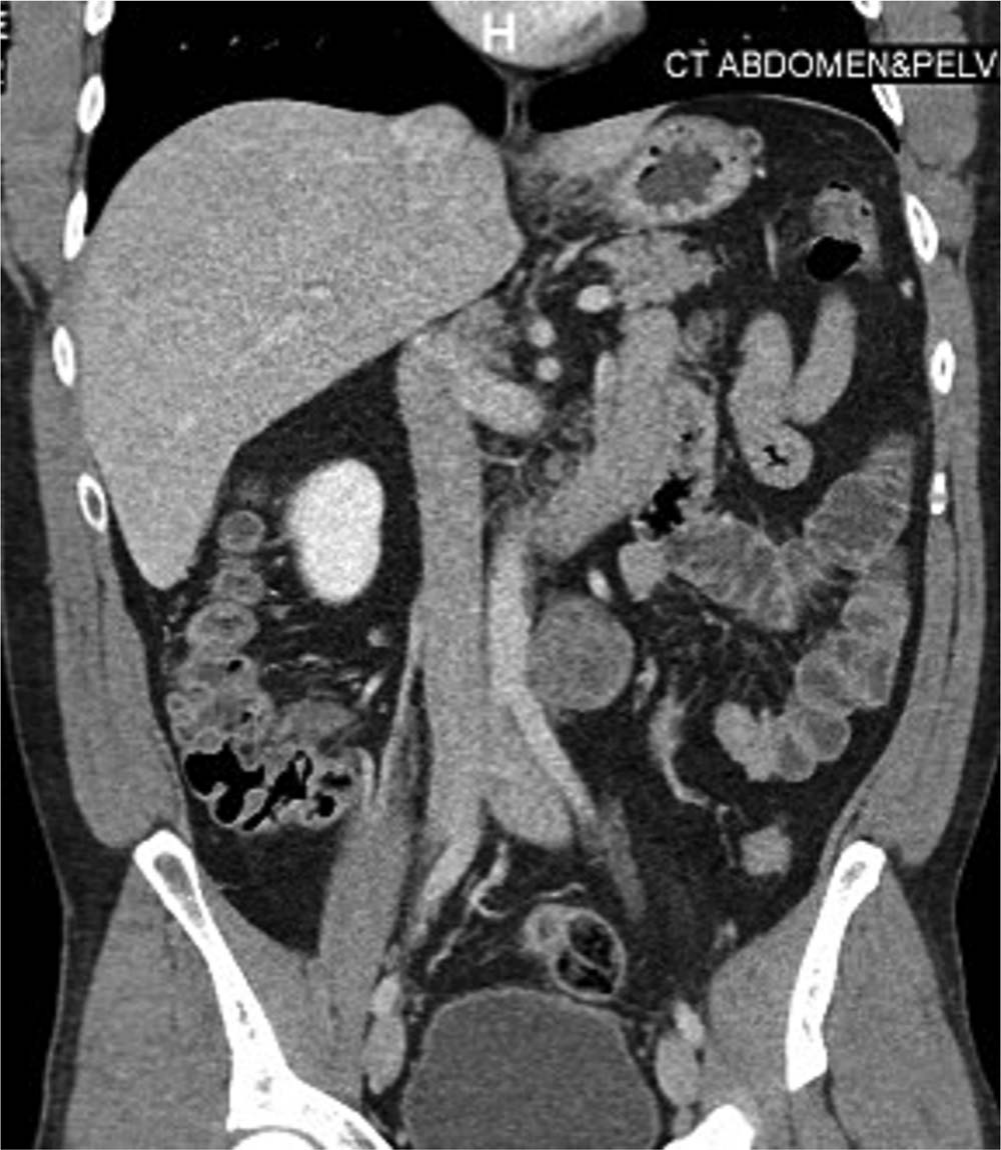
Initial CT coronal.

**Figure 3:**
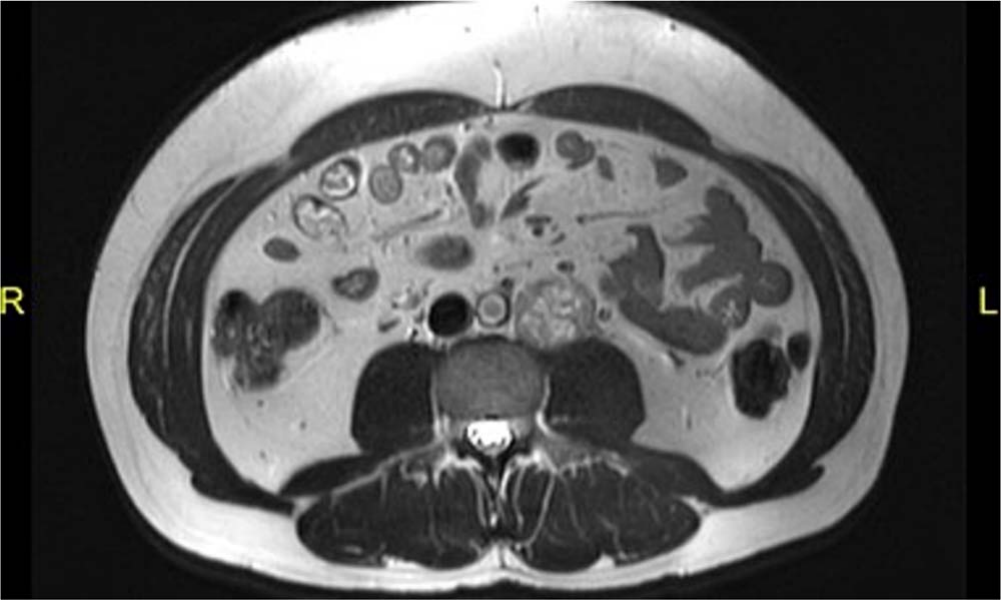
MRI coronal.

**Figure 4:**
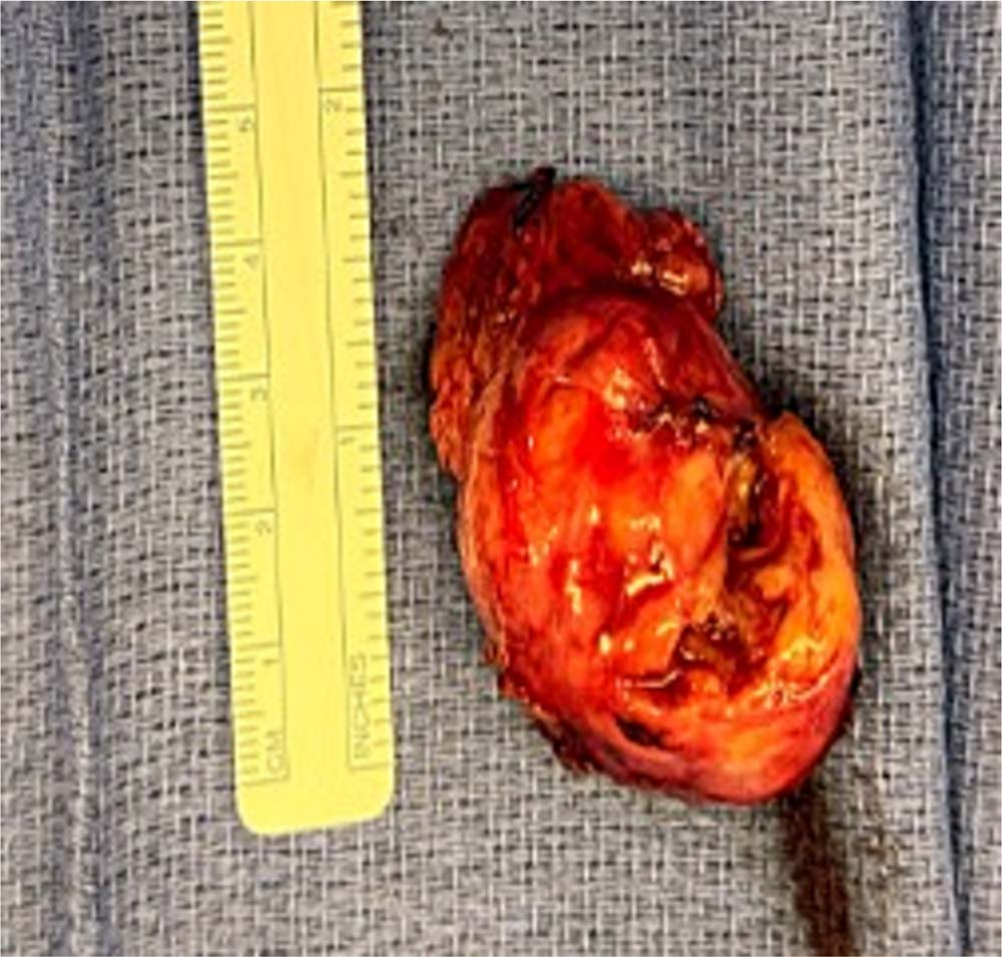
Specimen.

**Figure 5:**
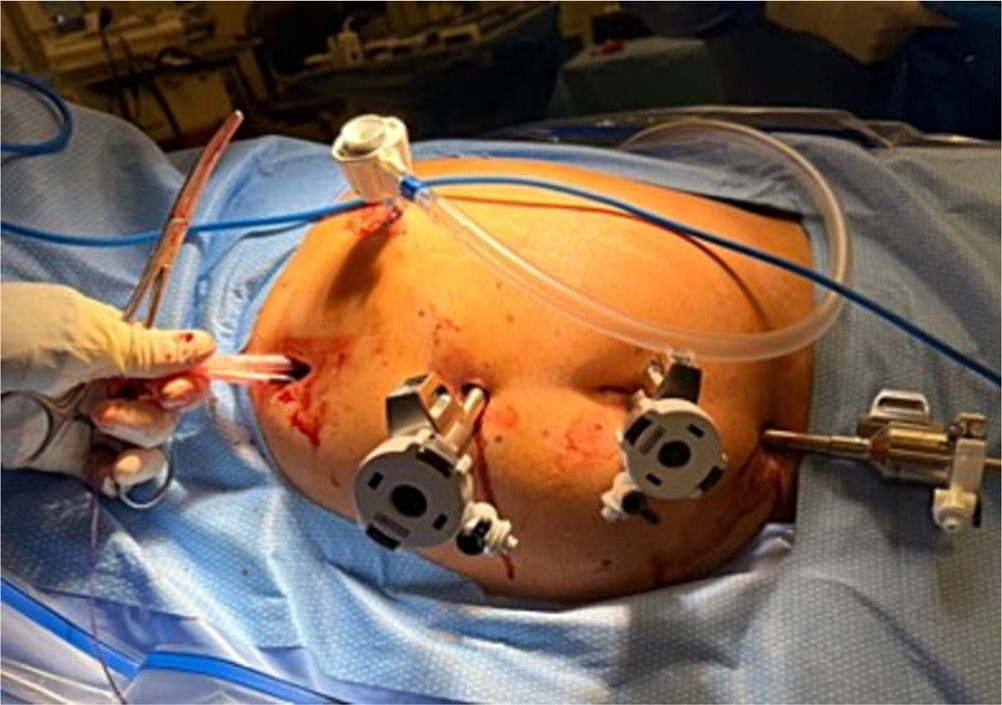
Trocar port placement.

**Figure 6:**
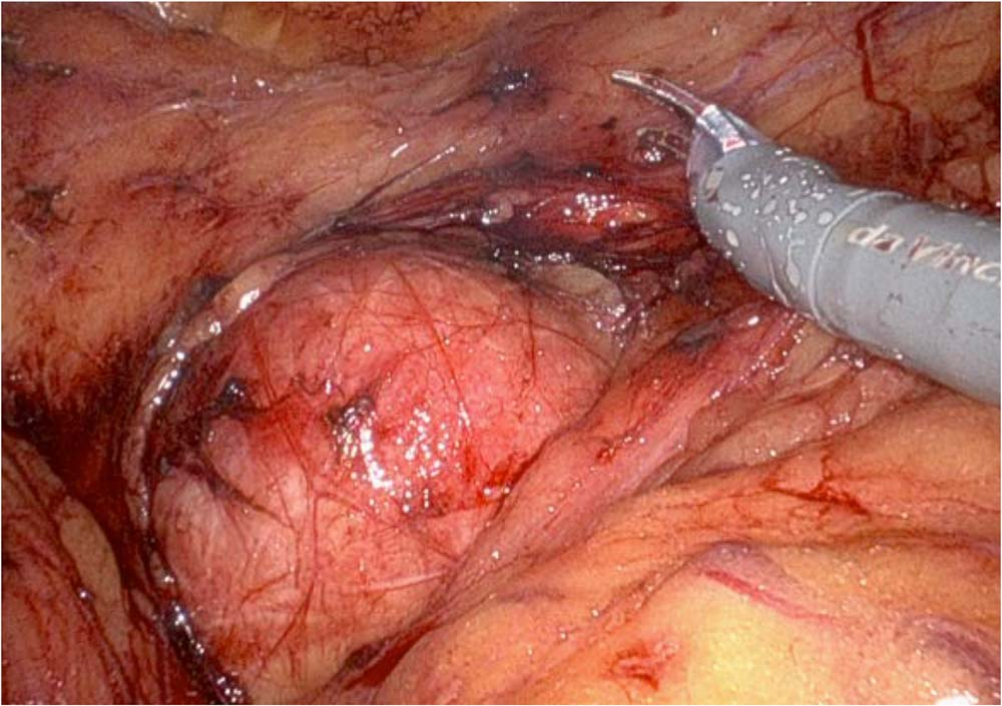
Intraoperative schwannoma.

**Figure 7:**
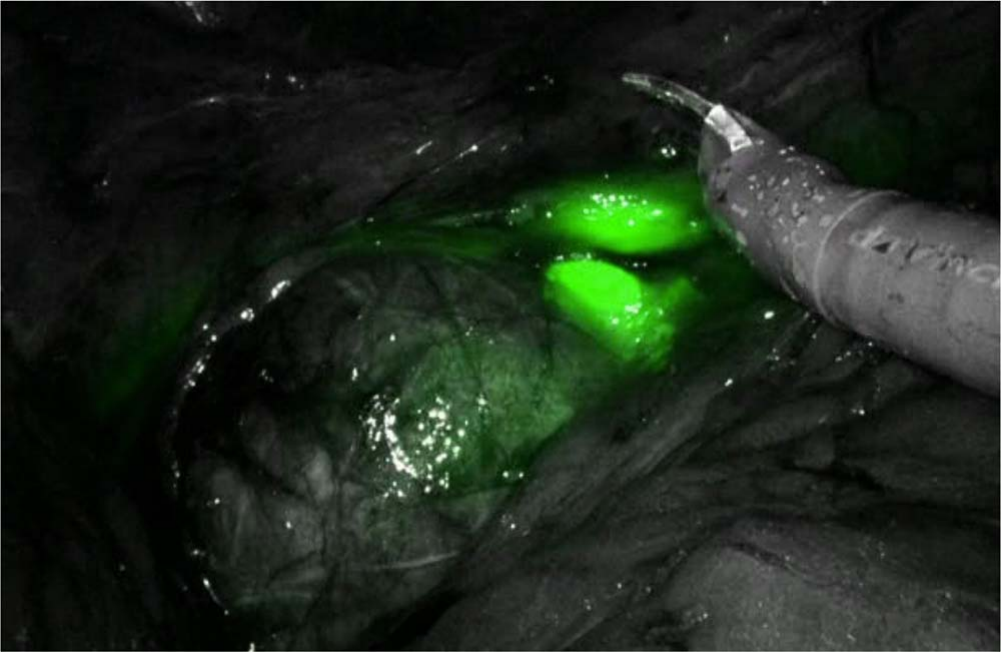
Intraoperative firefly use in identification of ureter.

A 41 year old male with a medical history of diabetes presented to the emergency department with a 2 week history of isolated right sided flank/abdominal pain. A computed tomography (CT) of the abdomen/pelvis was obtained and a well-demarcated spherical mass in the inferior left retroperitoneum, peri measuring 3.4 cm in maximum transverse diameter was discovered (Figs 1 and 2). He was referred to hematology-oncology; and 6 weeks later, he underwent MRI as well as CT-guided IR biopsy of the mass, which came back positive for schwannoma without evidence of lymphoproliferative disorder. Repeat imaging demonstrated a stable appearance of the mass (Fig. 3). He was then referred to surgical oncology. He underwent a robotic assisted retroperitoneal complete mass excision utilizing a modified Mattox maneuver to get exposure as well as preoperative ureteral stenting (Figs 4 and 5). During the case, the stents laced in the ureters to help identify and locate during the case given the close proximity as identified on the preoperative CT scans (Figs 6 and 7). Utilization of both blunt dissection and electrocautery, the entire mass was dissected free without any damage to ureter or aorta. He discharged home on post-op day 1 and was doing well on initial post-op check.

## Discussion

Specific challenges associated with this para-aortic and para urethral mass were the safe yet complete excision of the mass without injury to surrounding structures. While the ureteral stents helped identify the location and course of the urethra, care had to be taken while manipulating the urethra to avoid tissue damage leading to potential devascularization or leaks. When dealing with the abdominal aorta, particular attention was drawn to both major and minor branches. Both the aorta and urethra are susceptible to thermal injuries and increased care had to be applied while using energy.

In conclusion, successful surgical resections start with optimal planning. In this case, patient and port positioning along with preoperative stent placement were crucial in obtaining complete resection without damage to nearby structures. This case highlights the importance of preoperative planning to optimize surgical outcomes.
